# Addressing coloniality of power to improve HIV care in South Africa and other LMIC

**DOI:** 10.3389/frph.2023.1116813

**Published:** 2023-03-29

**Authors:** Claudia E. Ordóñez, Vincent C. Marconi, Lenore Manderson

**Affiliations:** ^1^ Department of Global Health, Rollins School of Public Health, Emory University, Atlanta, GA, United States; ^2^ School of Medicine, Emory University, Atlanta, GA, United States; ^3^ School of Public Health, University of the Witwatersrand, Johannesburg, South Africa

**Keywords:** HIV care, community-based stakeholder engagement, community-engaged research, South Africa, coloniality of power, care integration, LMIC, implementation science

## Abstract

We describe the appropriateness and potential for effectiveness of three strategic approaches for improving HIV care in South Africa: community-based primary healthcare, local/community-based stakeholder engagement, and community-engaged research. At their core, these approaches are related to overcoming health inequity and inequality resulting from coloniality of power's heterogenous structural processes impacting health care in many low- and middle-income countries (LMIC). We turn to South Africa, a middle-income country, as an example. There the HIV epidemic began in the 1980s and its ending is as elusive as achieving universal healthcare. Despite impressive achievements such as the antiretroviral treatment program (the largest in the world) and the country's outstanding cadre of HIV experts, healthcare workers and leaders, disadvantaged South Africans continue to experience disproportionate rates of HIV transmission. Innovation in global public health must prioritize overcoming the coloniality of power in LMIC, effected through the imposition of development and healthcare models conceived in high-income countries (HIC) and insufficient investment to address social determinants of health. We advocate for a paradigm shift in global health structures and financing to effectively respond to the HIV pandemic in LMIC. We propose ethically responsive, local/community-based stakeholder engagement as a key conceptual approach and strategy to improve HIV care in South Africa and elsewhere. We join in solidarity with local/community-based stakeholders' longstanding efforts and call upon others to change the current status quo characterized by global public health power concentrated in HIC.

## Introduction

The 1978 Global Conference on Primary Health Care (PHC) produced the Alma-Ata Declaration on achieving health for all by the year 2000. Forty years later, the Astana Declaration reaffirmed commitment to this goal, while renewing attention to the persistence of health inequalities and the unfulfilled need to implement the core principles of Alma-Ata. The Astana Declaration also called attention to PHC as critical in achieving health-related targets of the Sustainable Development Goals (SDG), which were adopted in 2015 by the United Nations General Assembly. The SDG include ending the HIV/AIDS epidemic by 2030 (SDG3 Target 3.3) and achieving universal health coverage (UHC) for all (SDG3 Target 3.8) (1).

Alma-Ata's central goal of “health for all” was the product of a global shift in political and social order connected to Western European colonial rule ending globally, especially in Africa. This shift brought to the forefront the devastating impact of former colonial rule and post-colonial relations on the health of oppressed, colonized populations, and the urgent need to address health inequalities produced by colonization. Under colonial rule, major disruptions in traditional community structures left indigenous populations without the economic and social support necessary to confront devastating endemic infectious diseases (e.g., malaria), new epidemics were unleashed from external sources (e.g., 1918 influenza pandemic), and large numbers of displaced individuals and famine contributed to periodic epidemics of local pathogens (e.g., African trypanosomiasis) (2–4). Very limited health services were concentrated in large cities where the minority of local populations resided, and mostly focused on interrupting transmission of infection. The treatment of disease among local indigenous populations was neglected, subsequently stigmatizing them by characterizing them as threats to public health and security (5, 6). These historical factors led to healthcare deserts and social determinants that predisposed further disparities.

The concept of *coloniality of power* was developed by Peruvian sociologist Aníbal Quijano during the latter part of the 20th century (7). It is defined as “a conceptual apparatus to apprehend the racial, political economic, social, epistemological, linguistic and gendered hierarchical orders imposed by European colonialism that transcended “decolonization” and continue to oppress in accordance with the needs of pan-capital (i.e., economic and cultural/symbolic) accumulation” (8). Globally, the 1980s saw international aid and development initiatives dominated by neo-liberal macro-economic and social policies. Structural adjustment programs were implemented which accentuated rather than reduced social inequality and inequity in low- and middle-income countries (LMIC). Austerity measures, aimed at reducing budget deficits through devaluing local currency and cutting public spending, particularly impacted health systems and social care programs, which became default areas for requisite cuts. Neo-liberal approaches brought new forms of oppression and consolidated the coloniality of power in the neo-colonial era. Resulting socioeconomic conditions and structural violence proved favorable to the spread of the human immunodeficiency virus (HIV) in LMIC, particularly in Southern Africa and especially in Botswana, Eswatini, and South Africa.

Leveraging our experience in medical anthropology and biomedicine to address biosocial aspects of the HIV epidemic in South Africa, we describe key HIV care challenges in neo-colonial LMIC; impacts of coloniality of power in South Africa's health system; and three strategic approaches with potential for improving HIV care in South Africa and addressing coloniality of power: community-based PHC (health system level), local/community-based stakeholder engagement (civil society level), and community-engaged research (CEnR) (global health implementation level). Given its prominence in the global health stage and our professional experience in the country, we chose South Africa as an example of HIV public healthcare in post-colonial LMIC. In our discussion and conclusion, we propose coloniality of power as an overarching conceptual framework to advocate for increased critical reflexivity and action to address the coloniality of global health interventions among powerful global health stakeholders/funders. We also advocate for using stakeholder engagement (SE) as a key conceptual strategy for effective and sustainable HIV care improvement in post-colonial contexts.

## HIV care in neo-colonial LMIC

LMIC consistently need to address the multiple challenges that impact population health: prioritization of health problems; supply of and access to goods, services, and human resources; and areas of research and investments pursued. Colonialism and globalization have appropriated resources and exploited labor in poor countries, and low wages, economic precarity, and poor living conditions all influence people's vulnerability and capacity to prevent infection and diseases. Inequalities and sustained poverty influence when and where people seek and receive ongoing care and the quality of that care. The continued transmission of infectious diseases and the growing burden of non-communicable diseases (NCDs) in LMIC (9) reflect the inability of countries to address the underlying social and commercial determinants of disease (10, 11), resulting in continued vulnerabilities that place people at risk of different infections, including HIV.

The deepened and sustained health inequalities today make the driving principles of the Alma Ata declaration as relevant as they were in 1978. Wealthier post-colonial middle-income countries, as assessed by gross domestic product, in general have greater income inequality; South Africa is the most unequal country in the world. Marginalized and vulnerable populations, including persons with HIV (PWH), continue to be left behind and seen as the “target” populations of weak PHC efforts. Despite the important global successes of HIV interventions (in South Africa: 45% decrease in HIV incidence between 2010 and 2020, 7-fold increase in the percentage of PWH who are on antiretroviral treatment, and >65% reduction in HIV-associated mortality (12)), poorer, marginalized, and oppressed populations in LMIC (as well as in high income countries) continue to experience disproportionate rates of HIV transmission (13).

Demonstrating a Western technocratic worldview, the approach of higher-income countries to eradicating HIV is “Global North-centric, managerial, data-driven, and biotechnological” (14), often ignoring structural inequities. Global public health funders and technocrats have focused on measurable, discretely identifiable outcomes (e.g., disability-adjusted live years or DALYs) as a measure of impact, which has ultimately driven health investments but has fallen short of more comprehensive health benefits. To realize WHO's aim for UHC as the key catalyst for improving health equity, it is necessary to transition from HIV selective or targeted and vertical approaches to more integrated health system responses (15). In other words, we need to continue to move further away from one-sided technocratic intervention models targeting cost-effective, short-term results, to interventions that support democratic, integrated, long-term approaches within sustainable healthcare systems. The integration of single disease health programs at the PHC level will empower countries to provide holistic healthcare.

## Intertwined structural and institutional shortcomings in South African healthcare

South Africa, an upper middle-income country (per World Bank 2022–2023 classification), provides an example of coloniality of power resulting in intertwined structural and institutional shortcomings. Under the apartheid system (1948–1993), which strengthened the racial segregation begun under colonial rule, the South African health system was characterized by racism and geographic disparities, fragmentation, duplication, and disproportioned focus on tertiary care while deprioritizing PHC (16). Challenges in delivering quality care persist in the post-apartheid era despite strong protection provided by the 1996 South African Constitution for the rights of all citizens and residents to access quality healthcare.

Presently South Africa deals with an extraordinary burden of disease and critical health system vulnerabilities. This includes a dual HIV and tuberculosis (TB) epidemic “(about 17% of global burden), high maternal and child mortality (about 1% of global burden), high levels of violence and injuries (about 1.3% of global burden) and increasing NCDs (about 1% of global burden)” (17). Public health system vulnerabilities include its aging and frail infrastructure, a substantial shortage of staff and resources, unequal distribution of resources, management and leadership crisis, negative staff attitudes, long waiting times, unclean facilities, medicine stock-outs, insufficient infection control, compromised safety and security of both staff and patients, pull and push factors, and slow healthcare system restructuring (18, 19). In this context, the health system's capacity to appropriately support and manage multiple health programs (e.g., integrated healthcare) at the PHC level is limited, particularly in marginalized and rural areas (20).

South African health policy has attempted to counter the impact of coloniality of power on the system. The 1996 “Integration of Services Policy” aimed to make PHC services more accessible and improve the efficiency of health service delivery through an integrated care model. The goal of integration remains elusive, however, while the need for equality on access to healthcare continues to be pressing with a 27% unemployment rate and 80% of 55 million South Africans relying on the public sector for healthcare (21). Recently, the National Health Insurance (NHI) initiative was launched to reduce inequalities by improving access and quality of care for all through expanded coverage. It remains unclear whether the NHI initiative will be able to overcome health system challenges and eventually achieve the goal of UHC. Presently, South Africa continues to strive toward services integration given SDG policy (22) and compelling evidence from the experience of integrating TB and HIV services, which has resulted in the reduction in HIV and TB-associated mortality and morbidity (23).

## Three strategies to support South Africa's care integration

We propose local/community-based SE, Community-based PHC, and CEnR as complementary strategies for the effective and sustainable integration of health services and improvements in HIV care. Adopted as an implementation strategy by the 1996 Integration of Services Policy, community-based PHC already uses local/community-based SE. The synergy between these two strategies could be furthered by using CEnR to design, deliver, and evaluate health services. This triad can address coloniality of power in healthcare by effectively promoting a democratic, less hierarchical approach to key health interventions such as care integration. Below we define and describe each of these strategies, starting with the one we consider to be indispensable in healthcare services and research: local/community-based SE.

### Local/community-based stakeholder engagement

In healthcare SE plays key roles as an *ethical approach to* and a *methodological strategy for* HIV care research and improvement and for care integration. We define stakeholder as any person or group of persons “who is responsible for or affected by health- and healthcare-related decisions that can be informed by research evidence” (24). Stakeholders are diverse, and may include patients, government health officials, local and international health experts and researchers, health activists, community engagement leaders from health non-profit sector, traditional health practitioners, and community health workers.

We understand SE in healthcare as the involvement of people who have a stake in health, those affected by or those who can affect decisions informed by research evidence. SE involves multiple communities and people in different intersecting positions of power, and comprehensive SE can successfully help to leverage local resources and community support of programs while addressing concerns related to agency, needs, and trust of the people receiving healthcare. Health ethicists have also argued that SE is a necessary component of global health and HIV research seeking health equity (25) and that ethical goals of engagement should include a) generating research topics and questions that reflect communities' needs, and b) promoting research translation into tangible benefits to communities (26).

Our approach to SE in this article is localized since effective community engagement (CE) is shaped by local conditions and culture (27, 28). We use “local” when referring to SE to denote stakeholders who are based in the same country (or region in some contexts) where the community of interest is located (i.e., civil society), as opposed to other possible stakeholders who may be outsiders to the country or region. Community-based stakeholders are local and based in the community of interest (i.e., people at the grassroots within communities), while local stakeholders do not need to be grassroot.

Different types of theoretical frameworks or approaches have been used to advance SE, usually falling in two general rationales or “meta-narratives.” A *health services* or *utilitarian perspective* understands SE as a tool to achieve more acceptable and appropriate health interventions, while the *social justice perspective* emphasizes empowerment and development of communities (29). In practice, the utilitarian and social justice rationales often merge, providing a balance between the interests of the public health stakeholders and communities.

SE is beneficial to health interventions and possibly necessary for achieving and sustaining health equity (29–31). In the context of LMIC, a 2017 review of systematic reviews on “the effectiveness of community engagement and participation approaches in LMIC” found moderate to limited strength evidence of these approaches being viewed as important in LMIC healthcare settings and playing a role in successful health intervention delivery (32). This review also found that achieving community ownership and empowerment greatly impacts on sustainability of engagement and participation (32). Although the strength of the effectiveness of CE evidence in this review was “moderate to limited,” and despite the lack of a more coherent body of evidence about the nature of CE and its contributions to performance of science programs in LMIC (33), CE appears to be decisive in improving performance of some science programs (34).

Interactions between global public health programs and stakeholders include securing access and permission, seeking cooperation and collaboration, fulfilling regulatory and ethical requirements, and shaping research strategies and the translations of their findings into policy or practice (34). However, these interactions can often be “motivated disproportionately by the interests and goals of the scientific programs and less by the need to elicit and understand their implications for stakeholders” (34). Researchers argue that more support is needed from funders, technocrats, and health system leadership to transcend from limited engagement that informs and/or consults with local stakeholders (seen often as tokenism), to a mode of engagement that enables and promotes higher levels of participation (involvement, collaboration, and empowerment levels) (35, 36), with potentially higher effectiveness and sustainability (29).

### Community-based primary health care

The community-based PHC model plays a key role in care integration in South Africa. The National Health Act 61 of 2003 formalized local community participation in PHC by mandating the establishment of health committees at all PHC facilities (37). However, this strategy has limitations due to inadequate provision of responsibility and authority and the lack of capacity support to the health committees linked to clinics (38).

The effectiveness of community-based PHC in Africa is tied to political commitment to and inclusion of Community-Oriented Primary Care (COPC) in policy (39). The COPC approach (a variation of Community-based PHC guided by data gathered within the community) was pioneered in 1940s rural South Africa and enabled the strong integration of primary health, community medicine, and CE. Care integration has been key in re-engineering South Africa's UHC and PHC strategies (40), and is perceived as compatible with Community-based PHC.

Integration of HIV, TB, and PHC in South Africa has been welcomed, with comprehensive case management, better client-nurse interactions, and reduced stigma all perceived as benefits of integration by stakeholders, notwithstanding challenges in terms of staff workloads and waiting times (20). One South African study showed that community health workers “managed to easily move from a HIV/TB focus to providing a comprehensive range of services across health and social conditions” (40). Although limited information is available to understand community involvement in priority setting, planning and decision-making in development and implementation of services, stakeholder participation in PHC has emerged as of great importance to realize UHC and the NHI (38).

### Community engaged research

CEnR is the umbrella term used for many different participatory approaches and methodologies, covering a spectrum of research approaches driven by the level of community inclusion and engagement, including community-based participatory research (CBPR) – the most cited approach (41).The origins of CEnR in Western academia date back to the 1940s with Kurt Lewin (northern tradition) and later Paulo Freire and Orlando Fals Borda (both representing the southern tradition) (42). CEnR has been recognized as key in all aspects of the process needed for developing and implementing programs and interventions in health (38) and to overcome health disparities (43). Yet, the historical structures and processes of academic health centers, including complexities of review boards operation, accounting practices and indirect funding policies, and tenure and promotion path, are a major barrier toward a translational CEnR agenda (43).

Longstanding partnerships between academia and communities impacted by HIV have paved the way for CEnR in HIV (44). In South Africa, CEnR has been a logical approach given the country's history of anti-apartheid activism and participatory ethos of political change, with many communities embracing participatory principles of collective action and mobilization (45). “Training for Transformation” is an example of a critical pedagogy participatory research methodology developed in the anti-apartheid context (46). There have been a significant number of HIV studies using participatory approaches and methodologies in South Africa in the past decade, addressing issues such as inequality and cultural differences, HIV drug adherence, welfare plans, and reduced HIV incidence (47–50). Nonetheless, barriers to the implementation of CBPR and good participatory practices continue and the extent of participation is highly variable, causing CEnR to be “often partially or incompletely implemented” (44).

## Discussion and conclusion

Related healthcare integration efforts in South Africa show some degree of effectiveness and ample acceptance among stakeholders, as well as the importance of broad base SE. Regarding Community-based PHC, a sub-Saharan Africa review found that community healthcare workers are providing a variety of important services to community-based HIV care, despite challenges related to training, resources, and supervision (51). The engagement of local/community-based stakeholders has also been consistently identified as beneficial in health interventions, with a continental-level analysis identifying the need for broad base and multisectoral stakeholder consultation for effective development of human resources for health strategic plans (52).

### The future: endemic HIV, integrated healthcare, and epistemic freedom

Community-based health hinges on the input, expertise, and labor of community members. To achieve healthcare system improvement and equitable access to healthcare for PWH and all community members, meaningful, generalized, and sustainable SE, including of community-based stakeholders, is necessary. Biomedical research and implementation science cannot have a fully effective or sustainable impact on clinical outcomes, programmatic development, or public health measures without authentic CE. Too often scientists address concerns that are irrelevant to the community or test solutions that were not developed in partnership with community-based and local stakeholders. These same scientists then find themselves confused as to why so many interventions fail in the real world.

Strong and broad SE is also key for HIV care improvement as the virus and disease transition to endemic status (15). Stakeholder involvement, particularly of community-based members and local health experts, needs to promote higher levels of participation, including involvement, collaboration and empowerment (29) and have the standing of an ethics requirement. This will support challenging the coloniality of power, which persists in global public health interventions and global health education in HIC (53). Increased, sustainable participation can uplift the “epistemic virtue” of local, community-based stakeholders by acknowledging and accepting the value of their knowledges and experiences (e.g., traditional and indigenous health knowledges (18)). In other words, truly inclusive SE would also support Ndlovu-Gatsheni's (54) call for epistemic freedom in Africa.

## Conclusion

To approach healthcare and end the HIV epidemic (SDG3) holistically, we need to move beyond single disease approaches to integrated healthcare and ethically responsive SE, with emphasis on local (non-external) and community-based (grassroots) actors. Despite progress (34), there is still critical need for a paradigm shift toward a horizontal approach in global health structures and financing (e.g., PEPFAR, Global Fund, National Institutes of Health, Gates Foundation) to effectively respond to future endemic HIV, emerging infectious diseases, and the continued high levels of communicable and non-communicable chronic diseases. Health programs that emphasize particular diseases and strategies run a continued risk of failing to reach goals as specific agendas are set aside as new challenges emerge. We see Community-based PHC and CEnR as effective, appropriate, and practical strategies that, in synergy with local/community-based SE, can produce tangible results such as local communities influencing “problem framings, program goals, and other key decisions” (55).

Innovation in global public health must address overcoming the coloniality of power in LMIC effected through the imposition of development and healthcare models conceived in HIC in the interest of pan-capital accumulation. We propose coloniality of power as a conceptual overarching framework and local/community-based SE as both a key *ethical approach to* and a *methodological strategy for* HIV care research and improvement. Some concrete innovation steps are including more leaders from LMIC in central, key decision-making roles in top global health structures, calling funders to fully cover costs and time of CE, and requiring rigorous evidence of appropriate local/community-based SE and CEnR as condition for funding and renewal.

We join in solidarity with community-based and local stakeholders' efforts, and call upon others to change the current status quo characterized by global public health power concentrated in HIC.

## Data Availability

The original contributions presented in the study are included in the article/Supplementary Material. Further inquiries can be directed to the corresponding author.
