# Supplementary material for: Addressing coloniality of power to improve HIV care in South Africa and other LMIC
Source: Front Reprod Health. 2023 Mar 29;5:1116813. doi: 10.3389/frph.2023.1116813 (PMC10090665; doi:10.3389/frph.2023.1116813)
Supplement: Supplementary file 1 [file Table1.docx]

We propose coloniality of power as a conceptual overarching framework and local/community-based stakeholder engagement as both a key *ethical approach to* and a *methodological strategy* *for* HIV care research and improvement in South Africa and other low- and middle-income countries (LMIC). Local/community-based stakeholder engagement, community-based primary healthcare (PHC), and community-engaged research (CEnR) are described as strategic approaches for improving HIV care in South Africa. Further, we posit community-based PHC and CEnR as complementary strategies that in synergy with local/community-based stakeholder engagement can contribute to effective and sustainable integration of health services in the South African context.

The paper’s description and analysis contribute to the body of literature documenting the effectiveness of these strategies and approaches for HIV care in South Africa. We also advocate for higher levels of stakeholder participation in health interventions, particularly of those who are community-based members and local (non-external) health experts, as this increment could uplift the ‘epistemic virtue’ of local/community-based stakeholders by acknowledging and incorporating the value of their knowledges and experiences. In other words, we see truly inclusive stakeholder engagement as supportive of indigenous calls for epistemic freedom in Africa and elsewhere, effectively challenging coloniality of power in LMIC.

We offer solidarity to calls for overcoming coloniality of power in LMIC effected through the imposition of development and health care models conceived in high-income countries, and propose ethically responsive, community-based stakeholder engagement as an effective and appropriate strategy to innovate and improve HIV care.
